# Exploring Knowledge, Availability, Accessibility, and Utilization of Emergency Contraception in Ghana: A Scoping Review

**DOI:** 10.7759/cureus.104630

**Published:** 2026-03-03

**Authors:** Ofeibea Asare, Sandra C Osuagwu, Anvita Dixit, Angel M Foster

**Affiliations:** 1 Faculty of Health Sciences, University of Ottawa, Ottawa, CAN

**Keywords:** africa, contraception, emergency contraception, ghana, scoping review

## Abstract

In Ghana, emergency contraception (EC) was initially introduced in the form of combined oral contraceptive pills and has since expanded to include progestin-only formulations and other methods. Despite these advances, unintended pregnancy rates remain high, suggesting limited use of post-coital contraceptive methods.

We undertook this scoping review to understand what is known about EC in Ghana, with a focus on knowledge, availability and accessibility, and utilization. We used an established framework to identify, chart, and summarize relevant source materials. We identified 57 source materials for inclusion in our review. We found that most EC research focuses on specific sub-populations, including students, urban dwellers, and unmarried women. Knowledge of EC varies but is almost exclusively centered on progestin-only emergency contraceptive pills (ECPs), and information appears to be sourced from both formal and peer-based channels. While progestin-only ECPs are available, geographic barriers to access persist. Some Ghanaians use progestin-only ECPs as their only ongoing method of pregnancy prevention, while others use them alongside traditional and modern methods.

Although several EC modalities are available in Ghana, knowledge and use are almost exclusively related to progestin-only ECPs. Most studies have been conducted with students, urban populations, and unmarried women. Research documenting women's lived experiences with seeking and obtaining all modalities of EC, identifying ways to increase awareness about a range of methods, and understanding the experiences of those who use progestin-only ECPs as their primary pregnancy prevention strategy appears warranted.

## Introduction and background

“Modern” contraceptive methods were introduced in Ghana in 1969, and since then their utilization has steadily increased [1,2]. There is a high unmet need for contraception, particularly among young women, with an estimated contraceptive prevalence rate of 50.7% [3]. Modern contraceptive method utilization is lower among youth [4,5], at 49.7% among 15-19-year-olds and 41.6% among 20-24-year-olds [6]. Research indicates that the unmet contraceptive need is tied to lack of access to affordable methods, fear of side effects, partner opposition, and infrequent sexual activity [7]. There is also widespread reliance on “traditional” contraceptive methods such as withdrawal, periodic abstinence, and fertility awareness [8,9]. These patterns are cause for concern given the relationship between unmet contraceptive need and unintended pregnancy [10,11]. In Sub-Saharan African countries, including Ghana, recent efforts have focused on promoting policies and programs that support uptake of long-acting reversible contraceptive methods, but short-acting methods, including post-coital contraceptive methods, have been underemphasized [12].

Emergency contraceptives are medications and devices that are used after sex to reduce the risk of pregnancy. This class of contraception is particularly valuable for reducing the risk of pregnancy after coerced sex or after under-protected or unprotected consensual sex [13,14].

In 1996, the Government of Ghana introduced emergency contraception (EC) in the form of combined oral contraceptive pills into its National Family Planning program and later incorporated dedicated emergency contraceptive pills (ECPs). Progestin-only ECPs became available through donor support, and other modalities of EC, including ulipristal acetate (UPA) and the post-coital insertion of the intrauterine device (IUD), are now available in the country [15-17]. All of these EC methods are included in the National Reproductive Health Service Policy and Standards of 2014, which are accessible and available across most healthcare facilities [18]. Research indicates that while many Ghanaians are aware of EC, its utilization is hindered by insufficient information and misconceptions [19-23]. This underutilization is likely due to inadequate health education and promotion efforts related to EC [24,25].

A 2019 study found that approximately 37% of pregnancies in Ghana were unintended [26]. Two additional studies indicated that 30% of unintended pregnancies occur among women aged 15 to 24 years [27,28]. Another recent study found that about 35.3% of unintended pregnancies occurred among adolescents aged 10 to 17 years [29]. The relatively high rate of unintended pregnancy among young people in Ghana may suggest underutilization of EC.

Although research to date does not indicate the magnitude by which use of EC could address Ghana’s challenges with unintended pregnancy and unmet contraceptive need at the population level, documentation of the current state of knowledge about EC in Ghana could inform policy development, programmatic efforts, and future research priorities. These initiatives could include youth-friendly outreach, capacity building of providers, and public education campaigns. We undertook this scoping review to assess what information exists about EC in Ghana, with a focus on knowledge, availability, accessibility, and utilization of EC.

## Review

Methods

We adopted the framework published by Arksey and O’Malley [30] and modified by Levac and colleagues [31] to develop our scoping review protocol. We used the five-stage framework for our study: 1) Identifying the research question; 2) Identifying relevant sources; 3) Selecting sources; 4) Charting the data; and 5) Collating, summarizing, and reporting the results. We focused on identifying peer-reviewed publications, grey literature, and policy/regulatory documents published by key organizations and researchers. Our team used the Preferred Reporting Items for Systematic reviews and Meta-Analyses extension for Scoping Reviews (PRISMA-ScR) checklist to guide the reporting of our findings [32].

Eligibility Criteria

We included studies focusing on Ghana or individual regions within Ghana, as well as studies in the African context that included Ghana. We included EC without restriction as to modality. We included both peer-reviewed and grey literature, such as original research articles, reviews, commentaries, books and book chapters, reports, and policies. We limited the publication language to English. We considered any published materials between January 2000 and December 2022 (inclusive). We did not critically appraise source material, and we did not exclude publications based on quality, consistent with the framework outlined by Arksey and O'Malley [30]. We present our inclusion and exclusion criteria in Table 1.

**Table 1:** Inclusion and exclusion criteria for our scoping review on what is known about emergency contraception in Ghana

Inclusion criteria	Exclusion criteria
Geographic location of Ghana, including any cities, regions, or subdivisions within the country; Geographic location of Africa and West Africa, including Ghana	Geographic location other than Ghana
Emergency contraception (all modalities)	Contraception other than emergency contraception
Publications dated on/after January 1, 2000, through December 31, 2022, English only	Published before 2000 or after 2022, Non-English language articles and publications
All types of articles and reports (original research, reviews, commentaries, book chapters, conference proceedings, reports, etc.)	Media articles

Information Sources and Search Strategy 

To find relevant studies, we conducted searches in the PubMed, Excerpta Medica database (EMBASE), Scopus, and JSTOR databases. We chose these databases for their relevance to literature on EC in Africa, including Ghana. Additionally, we identified some literature resources using Google Scholar and relevant websites of international sexual and reproductive health (SRH) organizations in the country to source grey literature. We consulted the University of Ottawa librarian subject specialist to develop the search strategy for our review. We used Boolean operators [33] to combine the terms and concepts for our searches. We provide an example search strategy in Appendix A.

Study Selection

We undertook the study selection through a two-stage review process. Initially, two reviewers (OA and SCO) independently screened all titles and abstracts to identify eligible texts for full screening. The reviewers resolved any disagreements about eligibility through discussion with AD. AMF was available to resolve disputes, but none arose. OA then conducted a full-text review and extracted information from eligible articles and uploaded all search data results into Covidence (Veritas Health Innovation Ltd, Melbourne, Victoria, Australia) [34] to manage the screening process.

Data Collection, Charting, Synthesis, and Analysis

We entered key information extracted from data sources into a Google Sheets© file (Google LLC, Mountain View, CA, USA). We extracted information on publication type, article type, year, methodology, geographical region, objective, key findings, and name of author(s). We then reviewed the corpus of sources for categories of content and themes. This process allowed us to synthesize the results and characterize the body of relevant literature.

Results

PRISMA Outline

**Figure 1:**
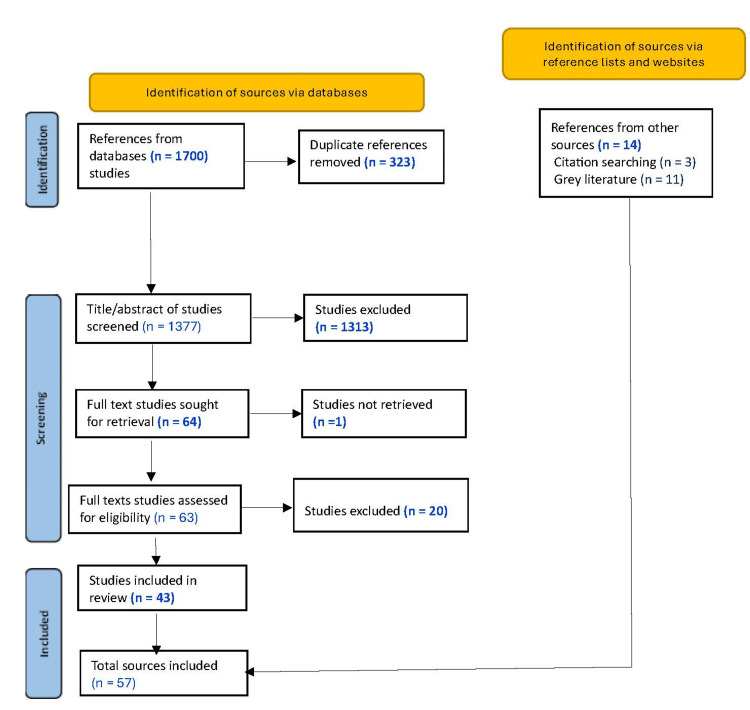
PRISMA-ScR flow diagram outlining the study selection process PRISMA-ScR: Preferred Reporting Items for Systematic reviews and Meta-Analyses extension for Scoping Reviews

Our searches identified a total of 1,700 articles from the databases. After removing 323 duplicates, we screened 1,377 sources on the basis of title and abstract. At this step, we excluded 1,313 sources, leaving 64 studies for full-text screening; we could not retrieve the full text for one of these sources, leaving a total of 63 eligible items. Of the 63 articles, we excluded 20 because they did not meet our inclusion criteria and used 43 in our review. A search through their reference lists yielded three additional sources for inclusion. Searching the websites of relevant organizations and networks resulted in a further 11 sources for inclusion. The number of materials included in our study totaled 57, as shown in Figure 1.

Characteristics of the Literature

We summarize the characteristics of the relevant literature in Table 2. Forty-three articles (75%) were retrieved from databases, and the majority were peer-reviewed articles (n=36); the remainder were student theses (n=2) and miscellaneous types such as a project evaluation paper, commentary, book chapter, study protocol, and review report (n=5). Most articles reported on quantitative studies (n=30), but we also identified qualitative (n=8) and mixed- and multi-methods (n=5) studies. We identified 14 sources (25%) through Google Scholar and SRH organization websites. The majority of our sourced materials (n=42, 73.7%) were solely from Ghana, and (n=15, 26.4%) were materials from international, Africa, and sub-Saharan Africa that included works from Ghana. Also, we identified that research on EC has expanded noticeably in the past decade, 2020 -2022, with substantially more studies published (n = 17, 30%) compared to the first 10 years of the review period.

**Table 2:** Characteristics of literature included in our scoping review on what is known about emergency contraception in Ghana (N=57)

Characteristic	Number
Type of source material	
Article in peer review journal	43
Other documents/websites	14
Research methods	
Quantitative	30
Qualitative	8
Mixed method	3
Multi-method	2
Other	14
Geographic focus	
Ghana (national)	42
Ghana and other countries	15
Year of publication	
2000 - 2003	4
2004 - 2007	3
2008 - 2011	7
2012 - 2015	14
2016 - 2019	12
2020 - 2022	17

Narrative Synthesis

A synthesis of the source material gives insight into the EC knowledge, availability, accessibility, and utilization in Ghana. We provide a summary of the published works on EC in Ghana in Appendix B.

Knowledge of EC in Ghana is variable: We found that Ghanaians have variable knowledge of EC; some studies reported high levels of knowledge, while others reported low or inadequate knowledge levels [35]. However, aside from progestin-only ECPs, knowledge of the other EC modalities, including IUDs, combined oral contraceptive pills, or UPA tablets, was virtually non-existent [36-41]. The literature indicates that Ghanaians obtain their EC information from a range of sources. These include health service providers, the media space (TV and radio), and relatives and friends/peers [36, 42].

A number of studies on EC were conducted among student populations, particularly in tertiary educational settings, to explore their knowledge [16, 39, 40, 43-51]. Taken together, these studies indicate that students from universities and secondary schools had relatively high awareness of progestin-only ECPs. Among university students, women demonstrated greater progestin-only ECPs knowledge than men. However, comprehensive knowledge was limited, and confusion over the effectiveness and optimal timing of use was considerable [43-45, 47-49, 52, 53]. Awareness of progestin-only ECPs also appears to be higher among university students, urban dwellers, and women of higher socio-economic status compared to their less educated, rural, and/or lower socio-economic status counterparts [5, 37, 54-56].

Four studies focused on EC knowledge among providers [57-60]. Taken together, these studies suggest considerable gaps in knowledge regarding post-coital insertion of the IUD and UPA. Knowledge about the timeframe for the use of progestin-only ECPs was generally accurate, but several studies noted that additional training should be offered to service providers in Ghana to improve overall awareness of EC [52, 59, 60].

Progestin-only ECPs are available in Ghana, but access barriers persist: Progestin-only ECPs have long been available in Ghana as both one and two-pill regimens [61]. They are offered in both public and private healthcare facilities, including Community Health Planning and Services (CHPS) compounds throughout the nation. Progestin-only ECPs are also available over the counter in pharmacies and licensed drug shops, and most users appear to obtain their pills from these service delivery points [7, 62-64]. EC-seekers can also obtain a post-coital IUD insertion at clinics and hospitals; both midwives and physicians can perform the insertion [65-67]. This may be especially important for those seeking a post-coital method after 72-120 hours [41, 68].

Findings from our scoping review provide mixed evidence about the availability and accessibility of progestin-only ECPs in urban versus rural areas. Some sources indicate there are no significant differences in availability and accessibility, while others show lower availability and accessibility in rural areas [36, 43, 60, 69, 70]. The literature also indicated that providers' attitudes, knowledge, and practices significantly influence users' access to progestin-only ECPs in Ghana [71]. Some sources made concrete recommendations about how to improve the availability and accessibility of progestin-only ECPs: implementing social marketing strategies directed at vulnerable populations and offering a free post-coital package that includes both progestin-only ECPs and external condoms [16, 71-73].

Utilization of progestin-only ECPs differs significantly by sub-population: Multiple studies have shown that young, sexually active, unmarried women aged 15 to 34 years are more likely to use progestin-only ECPs compared to their older, sexually active, married counterparts [19, 21, 44, 74-76]. The majority of these users reported progestin-only ECPs two to four times a year [21, 77]. Findings from surveys in the literature indicate that an insignificant minority of participants reportedly rely exclusively on progestin-only ECPs for pregnancy prevention. Others mentioned that they use ECPs alongside traditional and modern contraceptive techniques or as a “backup” [78-80].

The literature indicates that the reasons for using EC varied. Participants in surveys mentioned using progestin-only ECPs after contraceptive failure (especially withdrawal), difficulty taking daily oral contraceptive pills, and concerns regarding external condom breakage [55, 81]. The vast majority of participants in studies included in the literature reported using progestin-only ECPs immediately or within 72 hours of intercourse [48]; in one study, some participants reported using the pills before sex [69].

The source materials we reviewed suggest that EC-users have mixed feelings about the use of EC multiple times or as a primary method of pregnancy prevention; some were unconcerned about side effects, whilst others mistakenly worried that frequent use might lead to health issues such as infertility or cancer [77, 78, 82]. Users who used progestin-only ECPs “repeatedly” mentioned experiencing side effects such as vomiting, fatigue, delay, or distortions inthe menstrual cycle [36, 43, 83]. Two studies conducted in the Ashanti region found that some women are using other drugs as ECPs incorrectly and unreliably to prevent pregnancy after sex, including Primolut N (N tablet) [84, 85]. One study reported that women were using progestin-only ECPs to end established pregnancies [62]. Our findings indicate that further training for service providers and public awareness campaigns on approved EC modalities and their proper use are necessary [15, 18, 86, 87].

Discussion

Our scoping review aimed to explore and synthesize the existing literature to better understand what is known about EC in Ghana, focusing on knowledge, availability, accessibility, and utilization. The literature suggests that awareness of progestin-only ECPs is relatively high, but awareness of other EC modalities and comprehensive knowledge of EC is limited. Further, most research in Ghana has focused on sexually active, young, and unmarried women, primarily students in tertiary institutions, populations that may have higher awareness than their socio-demographic counterparts. Indeed, studies on EC knowledge across Africa have reported uneven knowledge among study participants [88-92].

Despite increasing attention to EC in Ghana, significant knowledge gaps remain across key sub-populations. Very limited research has examined EC awareness among out-of-school young people, and almost no studies have explored the knowledge of married individuals. Similarly, the perspectives of male partners on EC remain under-investigated, despite their potential influence on decision-making and access. Research on providers’ attitudes toward young clients at service delivery points is also warranted, as such insights could inform barriers to timely and equitable access. These gaps highlight the need for broader inquiry into EC awareness across diverse groups, with implications for the design of inclusive awareness-raising campaigns.

In addition, there is a pressing need to evaluate the effectiveness of awareness campaigns and training interventions through longitudinal studies. Longitudinal research assessing changes in utilization patterns and the persistence of misconceptions over time would provide an evidence base for policy and program design. For Ghana, another key priority is to integrate EC education into the broader framework of SRH education, thereby normalizing EC as a legitimate component of comprehensive family planning. Such integration would not only strengthen knowledge but also contribute to reducing stigma and ensuring equitable access across populations.

The existing literature indicates that progestin-only ECPs are widely available across Ghana through multiple service delivery points [93, 94]. However, disparities in both availability and accessibility persist between urban and rural areas. Evidence from sub-Saharan Africa further highlights that awareness of EC is consistently higher among urban and more educated women, while rural and less educated populations remain disproportionately less informed, reinforcing persistent knowledge gaps [37]. In rural Ghana, residents may encounter additional barriers due to shortages of healthcare professionals and a reduced number of service delivery outlets [95, 96]. These inequities are particularly consequential for women seeking care beyond 72 hours after unprotected intercourse, when the efficacy of progestin-only ECPs begins to wane. In such cases, UPA use and IUD insertion offer more effective alternatives, especially within the 73-120 hour window [97, 98].

Increasing awareness of the full range of EC modalities, particularly in rural and remote areas, is therefore critical. Also, strengthening access through the establishment of CHPS compounds and expanding the role of licensed drug shops and pharmacies could help bridge these gaps, aligning with recommendations from other Sub-Saharan African contexts [64, 99]. Despite these insights, there remains a paucity of research specifically examining EC access in rural Ghana, underscoring the need for further investigation into supply dynamics, referrals, cost, and service delivery in these underserved areas.

Consistent with research around the world [97, 100], women in Ghana report using EC for a variety of reasons, including contraception failure and as a “back-up” method [27]. However, research in Ghana also shows that a subset of women are using progestin-only ECPs as their primary, and only, contraceptive method. According to the International Consortium for Emergency Contraception (ICEC) and the World Health Organization, frequent use of progestin-only ECPs is effective, safe, and does not pose any potential adverse side effects, as highlighted in our study [86, 95, 96, 101, 102]. However, little is known about this subgroup of frequent users. Future research that explores the experiences of these women could not only lift up their voices but also provide information that could be used for advocacy and to dispel myths about “repeat” use [13, 103-105].

Service providers have been consistently identified as important sources of information on EC in Ghana [18, 42, 58, 65]. However, our findings indicate that some providers lack adequate knowledge of the full range of EC modalities. Tailored and strategic information campaigns targeting health service professionals could therefore enhance their engagement with clients and ensure the provision of accurate, evidence-based information [106, 107]. Similarly, training service providers on the comparative effectiveness of EC methods, their role in reducing unintended pregnancy, and the importance of youth-friendly counselling would not only improve service quality but also strengthen provider participation in community-level EC campaigns, thereby boosting client confidence.

At the same time, persistent myths, such as fears that EC causes infertility or even cancer, as noted in our work, continue to deter use and reinforce stigma. These misconceptions intersect with partner influence and entrenched gender norms. These often constrain women’s decision-making autonomy and further entrench misinformation. Addressing these sociocultural barriers alongside provider training is therefore essential to improving EC knowledge and uptake.

Nonetheless, efforts to expand awareness through providers or broader public campaigns may encounter policy-related barriers. Currently, Ghana’s national guidelines stipulate that EC should not be promoted as a “regular” family planning method [61, 86, 108]. Findings from this scoping review suggest that such a restrictive policy stance is short-sighted, as it risks contributing to misinformation, undermining knowledge and use, and limiting access. Instead, greater investment in awareness-raising, addressing gaps in provider knowledge, countering myths, and expanding accessibility is warranted to support informed uptake of EC across diverse populations.

Limitations

We aimed to use clear and consistent search terms to identify relevant literature for our review. However, we may have missed some sources. We intentionally decided not to critically appraise research articles, so we cannot reflect on the quality of studies included in our review. Additionally, we chose not to include media outputs in our search; journalistic accounts might provide more insight into women's lived experiences. Finally, we did not include the consultation phase in our scoping review process; engaging with experts may provide additional insights.

## Conclusions

Our scoping review provides insights into the current understanding of EC in Ghana, focusing on knowledge, availability, accessibility, and utilization. Our study revealed that knowledge and use were almost exclusively associated with progestin-only ECPs. Most studies focused on students, urban dwellers, and unmarried women, which may paint an incomplete picture. Future research is needed to explore the knowledge and experiences of other sub-populations. Women’s voices are also notably absent from the existing literature. More research documenting women's lived experiences with seeking and obtaining all modalities of EC, identifying ways to increase awareness about a range of methods, and understanding the experiences of those who use progestin-only ECPs as their primary pregnancy prevention strategy appears warranted.

## Appendices

Appendix A

Example Search Strategy for Scopus With Boolean Operators

(TITLE-ABS- KEY (''emergency AND contraception'')OR  TITLE-ABS-KEY(''emergency  AND contraceptives'')OR TITLE-ABS-KEY ( ''emergency  AND contraceptive  AND pills'' )  OR  TITLE-ABS-KEY(''post-coital  AND pills'')OR  TITLE-ABS-KEY("post-coital intrauterine device")OR  TITLE-ABS-KEY(''combined AND hormonal AND ec'') AND  TITLE-ABS - KEY (Ghana )) AND PUBYEAR > 1999 AND PUBYEAR < 2023 Results = 32

Appendix B

**Table 3:** Summary of eligible studies included in our scoping review on what is known about emergency contraception in Ghana (N=57)

N^o^	Author, Year, and Reference Number	Aim/Objective	Method	Country/Region	Key Findings
1	Addo VN et al., 2009 [43]	To investigate the emergency contraception (EC) knowledge, attitudes, and practices among students at a university in Ghana.	Quantitative	Ghana	Students have higher knowledge about progestin-only EC pills than oral contraceptive pills (OCPs) and intrauterine device (IUD) EC modalities.
2	Adjei KK et al., 2015 [65]	To assess the availability of modern contraceptive methods in public and private health facilities and factors affecting this, as perceived by facility managers in the Ga East municipality.	Mixed method	Ghana, Greater Accra	Progestin-only EC pills are available primarily in private health facilities such as pharmacies and licensed drug shops.
3	Amalba A et al., 2014 [36]	To investigate the awareness and use of ECPs among reproductive-age (15–49) women in Tamale, Ghana.	Quantitative	Ghana, Northern region	Women learn about ECPs from health workers, radio/TV, and family members/friends. About 85% know the correct timeframe for the effective use of ECPs to prevent pregnancy. Recognizing the need to take the first dose within 72 hours after having unprotected sexual intercourse.
4	Asiedu C et al., 2022 [44]	To assess the knowledge and perception of senior high school students about emergency contraceptive use in the Garu and Tempane Districts.	Quantitative	Ghana, Upper East Region	Students reported that they have heard about EC before, and they have both negative and positive perceptions about EC.
5	Asiedu C et al., 2019 [76]	To assess the utilization of contraceptives among women in their fertility age (15 – 49 years) living within the Effutu Municipality quantitatively.	Quantitative	Ghana, Central Region	Women were aware of EC. Despite the education on family planning, there were still some myths or beliefs people have about the use of contraceptives.
6	Awopegba OE et al., 2021 [37]	To investigate trends of EC among women in Sub -Saharan Africa (SSA), we limited the scope to 28 countries in SSA that have two survey years of information on EC awareness.	Quantitative	SSA, Ghana	EC knowledge level was lowest among adolescent girls in the countries studied. The knowledge of EC is high among Ghanaian women.
7	Baiden F et al., 2002 [16]	To assess knowledge and attitude toward EC among a sample of students at the University of Ghana.	Quantitative	Ghana	Respondents knew emergency contraceptive methods and Postinor-2 as a dedicated EC product on the Ghanaian market. Only 11.3% of respondents indicated the recommended time correctly, within which ECPs are to be taken after unprotected sex.
8	Bawah AA et al., 2021 [27]	To evaluate the impact of the Willows program.	Quantitative	Ghana, Ashanti Region	By the end of the project, an evaluation showed that women used EC and discontinued due to various factors such as the desire to get pregnant, side effects, method failure, and switched to other methods.
9	Both R, 2013 [35]	To review qualitative evidence from seven studies on young people’s experiences with this contraceptive (EC) method.	Qualitative	SSA, Ghana	Women in Accra who used EC thought the use might cause infertility, fibroids, or cancer. However, the majority were unconcerned and mentioned that they had not experienced any side effects from multiple use of EC.
10	Creanga AA et al., 2011 [57]	To assess the theoretical and practical knowledge about EC among family-planning (FP) providers in Ghana, and to examine the association between FP providers’ theoretical and practical knowledge.	Quantitative	Ghana, Ashanti Region	Family planning service providers, on average, gave correct answers to questions assessing their theoretical knowledge and correct answers to questions assessing their practical ability to provide EC.
11	Dam A et al., 2022 [68]	To systematically review the literature on contraceptive values and preferences of pregnant women, postpartum women, women seeking emergency contraception, and women seeking abortion services globally.	Quantitative	International, Ghana	A systematic review showed that nearly all participants preferred ECPs as their primary method of pregnancy prevention, often used with condoms and traditional methods.
12	Darteh EKM and Doku DT, 2016 [45]	To explore the extent to which university students in Ghana have knowledge of and use ECs, and explore the drivers of EC knowledge and use.	Quantitative	Ghana, Central Region	Although most of the respondents were sexually active, knowledge of EC, as well as its use, was relatively low among some of them.
13	Fuseini K et al., 2022 [75]	To use routine service data from the District Health Information Management System (DHIMS) to assess the impact of the COVID-19 pandemic on the use of ECs in Ghana	Quantitative	Ghana	Routine service data indicated increased EC use in 2020 due to the COVID-19 pandemic to prevent pregnancy.
14	Hallidu M and Sumaila I, 2022 [46]	To identify the determinants of ECs utilization among female tertiary students in the Middle Belt of Ghana, West Africa.	Quantitative	Ghana, Bono East	A study in the Bono East indicated that EC utilization among students was relatively low, although most students knew ECs.
15	Issah F, 2022 [47]	To investigate the knowledge, attitudes, and practices of emergency contraception use among female students at the University for Development Studies, Tamale campus.	Mixed method	Ghana, Northern region	Most of the study participants demonstrated sufficient knowledge of EC and had a “positive” attitude toward EC use.
16	Kalamar A et al., 2021 [77]	To explore societal and community norms, knowledge, behaviour, and attitudes in Accra, Ghana, and Lusaka, Zambia.	Qualitative	Greater Accra, Ghana, and Lusaka, Zambia	The findings from Ghana showed that ECPs are a trusted method and often preferred as an easy and effective option; secondly, people value ECPs as an on-demand method, yet fear that repeated use could have harmful health effects.
17	Konlan KD et al., 2020 [48]	To assess the utilization of ECs among female senior high school students in the Ho Municipality of Ghana	Quantitative	Ghana, Volta Region	The required time for EC to be taken was stated as immediately after sex, 24 hours after sex, and others did not know. Knowledge of EC was acquired from friends, family members, and the mass media.
18	Kpiinfaar TN et al., 2022 [38]	To determine factors influencing contraceptive use among adolescents in Techiman Municipality.	Quantitative	Ghana, Bono East Region	Contraceptive use among students was high, but a vast majority relied on non-long-acting reversible contraception (LARC) methods, including condoms and ECPs.
19	Krakowiak-Redd D et al., 2011 [85]	To generate an evidence base for potential (family planning) interventions in the Barekese sub-district.	Quantitative	Ghana, Ashanti Region	Some women misused Primolut N ("N- tablets") as EC. The most common reason women used it as EC was that they believed it was effective.
20	L'Engle KL et al., 2011 [78]	To investigate the potential for bridging ECP users who purchase the product in pharmacies to longer-term contraception.	Qualitative	Ghana, Greater Accra	ECPs occupied a central role in the participants' contraceptive method mix. Almost all participants agreed that ECPs were their primary method of pregnancy prevention. They were unconcerned about the repeated use of ECPs. Also, they had not experienced any side effects from repeated use, nor did they anticipate experiencing any problems in the future.
21	Lovvorn A et al., 2000 [80]	To evaluate the effect of two approaches to ECP provision on ECP use and unprotected intercourse among users of spermicide, a relatively popular choice among modern contraceptive method users in Ghana.	Quantitative	Ghana, Greater Accra, Eastern, Ashanti, and Western Regions	After the intervention, almost all participants thought ECPs were helpful for women and would recommend ECPs to their friends or relatives.
22	Machiyama K et al., 2018 [7]	To promote an evidence-based approach for improving access to family planning and safe abortion.	Mixed method	Ghana	Younger focus group participants emphasized their own and their friends' use of ECPs and discussed it in a way that suggested it was normalized in their peer groups.
23	Manortey S et al., 2016 [39]	To evaluate the awareness and use of ECPs among students in a tertiary institution in the Western Region of Ghana.	Quantitative	Ghana, Western Region	A high level of ECP knowledge among the student population was not matched by usage. They lacked detailed knowledge about the effectiveness and the timing schedule after unprotected sex.
24	Marston C et al., 2017 [82]	To understand how educated women in Ghana were achieving low levels of fertility with such low reported levels of contraceptive use.	Qualitative	Ghana	Women did not express concerns about the side effects of ECPs, although they said they worried about overuse. However, overuse and any harm were not defined. Women seemed to see ECPs as different from other hormonal methods, for which side effects were frequently mentioned.
25	Mayhew S et al., 2013 [58]	To understand providers’ knowledge of EC, their attitudes toward its use, and how they respond to users seeking care or help.	Qualitative	Ghana and Burkina Faso	In general, providers displayed knowledge of all EC modalities, especially ECPs. Most (12 in each country) had given EC in some form, primarily progestin-only or combined ECPs.
26	Mohammed S et al., 2019 [60]	To assess the knowledge, awareness, attitude, and use of EC and determine the association between these factors and the socio-demographic characteristics of female nursing and midwifery students of Tamale Nurses and Midwives Training College in Northern Ghana	Quantitative	Ghana, Northern Region	The service providers' trainees indicated they had heard about EC before the study. The majority of them correctly indicated the time within which to take ECPs. More than half did not know the appropriate time within which to use an IUD as EC.
27	Nachinab GTl et al., 2022 [51]	To investigate the utilization of EC among final year female students at the University for Development Studies (UDS), Tamale campus	Quantitative	Ghana, Northern Region	The students demonstrated sufficient knowledge and a favorable attitude toward EC. They identified when EC can be used: after unprotected sex, after a missed period, when one is raped, in an unwanted pregnancy, and rupture of condoms.
28	Nketia R et al., 2022 [42]	To examine the level of knowledge on EC, attitude towards EC, utilization, and barriers to EC among reproductive-age women between 15 and 24 years in the East-Gonja Municipality of Ghana.	Quantitative	Ghana, Savannah Region	The respondents had inadequate knowledge of EC. More than half reported prior knowledge of EC. Some respondents who indicated they had ever engaged in vaginal intercourse had ever used EC.
29	Opoku B and Kwaununu F, 2012 [69]	To look at the knowledge and practices of EC among women of reproductive age.	Quantitative	Ghana	The women knew EC. Some participants have used combined Pills, relied on the Postinor brand, and used Norethisterone. None of the participants had used an IUD as EC. Some have used EC before sex.
30	Opoku B and Kwaununu F, 2011 [84]	To look at the EC knowledge and practices of women of reproductive age in the country.	Quantitative	Ghana	The females knew about EC and had used ECPs, but did not know the IUD as EC.
31	Oppong FB et al., 2021 [5]	To assess the prevalence of contraceptive knowledge, use, and the determinants of contraceptive use among sexually active unmarried young women in Ghana.	Quantitative	Ghana	Knowledge of EC as a contraceptive method is almost universal among sexually active, unmarried adolescent girls and young women in Ghana. Of the 809 participants, 76.1% knew of EC, and only 7.2% used it.
32	Osei IF et al., 2014 [79]	To understand the social and relational contexts in which reproductive decisions are made.	Qualitative	Ghana, Greater Accra	Women mentioned EC as an infrequently used method, but occasionally relied on it when sexually active. Some mentioned they solely use EC, and others said they combined EC with traditional methods and condoms
33	Osei-Tutu E et al., 2018 [40]	To identify students’ depth of knowledge of EC and the extent to which they use ECPs	Quantitative	Ghana, Greater Accra	Knowledge and use of EC among tertiary students were limited. The majority were aware of some contraceptive methods, including ECPs. Despite this knowledge, the majority had never used any form of EC.
34	Osei–Tutu EM, 2019 [49]	To explore the relationship between male students’ knowledge of and attitude towards EC.	Quantitative	Ghana, Greater Accra	More respondents who know EC prevents pregnancy had mixed attitudes compared to those who disagreed. Thus, fewer of them had favorable attitudes compared to those who disagreed.
35	Osei-Tutu EM et al., 2018 [53]	To identify students’ knowledge of when to use EC in relation to sex. Identify the extent to which students/their partners use ECPs. Discover the reasons for the non-usage of ECPs by students.	Quantitative	Ghana, Greater Accra	Despite the knowledge of contraceptives, the majority had never used contraceptives, including EC.
36	Palermo T et al., 2014 [54]	To help fill important gaps in knowledge surrounding who knows about and is using EC globally to inform programmatic and policy recommendations	Quantitative	Multi-country, Ghana	In Ghana, those who had had some secondary or higher education, compared with those who had had less than a complete primary education, have used EC. Knowledge of EC was positively associated with education.
37	Rokicki S and Merten S, 2018 [21]	To understand the context and patterns of ECP use among young unmarried women in Ghana.	Qualitative	Ghana, Greater Accra	ECPs appear to be a popular contraceptive choice among young urban women in Ghana, yet misinformation about their correct usage and safety is widespread. Most participants have used ECPs at least once.
38	Steiner MJ et al., 2000 [59]	To evaluate the success of these early efforts to introduce EC into Ghana.	Qualitative	Ghana, Greater Accra and Ashanti	An evaluation found that providers’ knowledge about EC was. Out of 325 healthcare providers, two-thirds were unaware of any method that can be used after intercourse to reduce the risk of pregnancy aside from progestin-only pills and Yuzpe.
39	Strong J, 2021 [66]	To understand men’s masculinities and their relationships with EC and abortion	Multi-method	Ghana, Greater Accra	A study protocol stated that EC knowledge is increasing. Most women in the reproductive years (15 -49) have heard of EC. ECPs are part of the contraceptive mix in Ghana.
40	Thompson J et al., 2014 [67]	To examine how pregnancy prevention and management services feature within post-rape care (PRC) services in sub-Saharan Africa, vis-à-vis World Health Organization (WHO) guidance, and to make policy and programmatic recommendations for expanding access to these services.	Multi-method	SSA countries, Ghana	The PRC guidelines and guidelines for RH/FP specify the needs of rape survivors, although many contain general provisions on emergency contraception. The protocols in Ghana indicated EC may be given up to 5 days/120 hours after rape.
41	Williams K, 2011 [71]	To identify and describe existing provider-related barriers to accessing EC in developing countries, as well as the prevalence of these barriers and documented research programs to address them.	Quantitative	Developing countries, Ghana	In the review report, providers’ knowledge, attitudes, and practices affect women’s access to EC.
42	Yeboah DS et al., 2022 [74]	To assess the factors influencing the use of emergency contraceptives among reproductive-age women in the Kwadaso Municipality, Ghana	Quantitative	Ghana, Ashanti Region	EC use among the women was high following unexpected, unprotected sex and when coitus interruptus failed.
43	Ziba FA and Yakong VN, 2019 [50]	To investigate the knowledge and utilization of EC among tertiary students in the Bolgatanga Municipality of the Upper East Region of Ghana.	Quantitative	Ghana, Upper East Region	Sexually active women had more knowledge of EC than those who were not. Those who have heard of EC have used it more than once.
44	Baiden F, 2005 [72]	N/A	Narrative - correspondence	Ghana	A correspondent on increasing access to EC indicated that the use of pre-packaged EC plus condoms could enhance the social acceptability of promoting EC access among vulnerable groups.
45	Parker C, 2005 [52]	N/A	Review –working paper	International, Ghana	A review paper mentioned provider knowledge of emergency contraception in Ghana; a third had heard about ECPs, and none of the providers had sufficient knowledge to prescribe it correctly.
46	Hossain Sharif M et al., 2009 [62]	N/A	Handbook	Developing countries, Ghana	A handbook on introducing and mainstreaming the provision of emergency contraceptive pills in developing countries.
47	Maries Stopes International – Ghana, 2021 [83]	N/A	Blog article	International, Ghana	A blog said ECPs do not inhibit implantation of a fertilized egg and should be used as abortifacients. However, ECPs are not harmful to pregnant women.
48	Ghana Statistical Service (GSS), Ghana Health Service (GHS), and ICF International, 2014 [56]	N/A	Report	Ghana	GSS reported that 64.1% of women and 63.7% of men in Ghana have knowledge of EC.
49	GHS, 2014 [18]	N/A	National Policy and Standard document	Ghana	A national policy document showed approved and dedicated EC modalities in Ghana: levonorgestrel pills, combined oral pills, Copper T, and Mirena intrauterine device. The policy stated that ECPs should not be promoted as a regular contraceptive method.
50	Ghana Health Service and UNFPA, Ghana, 2015 [64]	N/A	Meeting Report	Ghana	A Ghana Health Service report stated that 98% of women and 99% of men know at least one method of contraception, including IUD, condoms, oral pills, implants, sterilization, injectables, and emergency contraceptives.
51	United States Agency for International Development (USAID), World Health Organization Regional Office for Africa (WHO/AFRO), Advance Africa, Advancing Women’s Access to Reproductive Health (AWARE-RH), and the POLICY Project, in collaboration with the Ghana Ministry of Health, United Nations Population Fund (UNFPA), International Planned Parenthood Federation (IPPF), and other collaborators, 2005 [63].	N/A	Report	International, Ghana	Policy-directed methods such as emergency contraception must be made available over the counter.
52	International Consortium for Emergency Contraception (ICEC), 2013 [73]	N/A	Fact sheets	International, Ghana	An ICEC fact sheet stated that Ghanaians have knowledge of EC and use it.
53	ICEC, 2017 [86]	N/A	Report	International, Ghana	64% of Ghanaians know EC and some have never used it.
54	ICEC & RH supplies, 2012 [61]	N/A	Fact sheets	International, Ghana	A project report stated that the gradual increase in EC provision can be attributed to the educational programs and campaigns done during the inception of their EC project in Ghana.
55	The Health Communication Capacity Collaborative HC3, 2014 [87]	N/A	Communication strategy document for emergency contraceptive pills	International, Ghana	A strategy to use EC to prevent unwanted pregnancies after sex and avoid unsafe abortions.
56	PATH Organization, 2003 [41]	N/A	A Curriculum for Young People in Africa, Ghana Version	Ghana	A curriculum on EC mentioned a special dose of OC pills that is taken within 72 hours of unprotected sexual intercourse. It is highly effective following: rape, contraceptive method failure, e.g., a broken condom, and after unprotected sex.
57	Population Council, 2021 [70]	N/A	Report	Ghana	EC can be accessed in all health facilities in both rural and urban settings. It accounts for 98% of contraceptive methods provided in Ghana.
